# The epidemiology of conjunctival squamous cell carcinoma in Uganda

**DOI:** 10.1038/sj.bjc.6600451

**Published:** 2002-08-01

**Authors:** R Newton, J Ziegler, C Ateenyi-Agaba, L Bousarghin, D Casabonne, V Beral, E Mbidde, L Carpenter, G Reeves, D M Parkin, H Wabinga, S Mbulaiteye, H Jaffe, D Bourboulia, C Boshoff, A Touzé, P Coursaget, the Uganda Kaposi's Sarcoma Study Group

**Affiliations:** Cancer Research UK, Epidemiology Unit, Gibson Building, Radcliffe Infirmary, Oxford OX2 6HE, UK; Uganda Cancer Institute and Makerere University Medical School, Kampala, Uganda; Laboratoire de Virologie Moléculaire, INSERM EMIU 00-10 and USC INRA, Faculté de Pharmacie, 37200 Tours, France; MRC Programme on AIDS, Uganda Virus Research Institute, PO Box 49, Entebbe, Uganda; International Agency for Research on Cancer, 150 Cours Albert-Thomas, Lyon, France; Centers for Disease Control and Prevention, 1600 Clifton Road, Atlanta, Georgia, GA 30333, USA; Wolfson Institute of Medical Research, University College London, 46 Cleveland Street, London, UK

**Keywords:** conjunctival carcinoma, HIV, HPV, KSHV, HHV-8, Uganda

## Abstract

As part of a larger investigation of cancer in Uganda, we conducted a case–control study of conjunctival squamous cell carcinoma in adults presenting at hospitals in Kampala. Participants were interviewed about social and lifestyle factors and had blood tested for antibodies to HIV, KSHV and HPV-16, -18 and -45. The odds of each factor among 60 people with conjunctival cancer was compared to that among 1214 controls with other cancer sites or types, using odds ratios, estimated with unconditional logistic regression. Conjunctival cancer was associated with HIV infection (OR 10.1, 95% confidence intervals [CI] 5.2–19.4; *P*<0.001), and was less common in those with a higher personal income (OR 0.4, 95% CI 0.3–1.2; *P*<0.001). The risk of conjunctival cancer increased with increasing time spent in cultivation and therefore in direct sunlight (χ^2^ trend=3.9, *P*=0.05), but decreased with decreasing age at leaving home (χ^2^ trend=3.9, *P*=0.05), perhaps reflecting less exposure to sunlight consequent to working in towns, although both results were of borderline statistical significance. To reduce confounding, sexual and reproductive variables were examined among HIV seropositive individuals only. Cases were more likely than controls to report that they had given or received gifts for sex (OR 3.5, 95% CI 1.2–10.4; *P*=0.03), but this may have been a chance finding as no other sexual or reproductive variable was associated with conjunctival cancer, including the number of self-reported lifetime sexual partners (*P*=0.4). The seroprevalence of antibodies against HPV-18 and -45 was too low to make reliable conclusions. The presence of anti-HPV-16 antibodies was not significantly associated with squamous cell carcinoma of the conjunctiva (OR 1.5, 95% CI 0.5–4.3; *P*=0.5) and nor were anti-KSHV antibodies (OR 0.9, 95% CI 0.4–2.1; *P*=0.8). The 10-fold increased risk of conjunctival cancer in HIV infected individuals is similar to results from other studies. The role of other oncogenic viral infections is unclear.

*British Journal of Cancer* (2002) **87**, 301–308. doi:10.1038/sj.bjc.6600451 www.bjcancer.com

© 2002 Cancer Research UK

## 

Squamous cell carcinoma of the conjunctiva is thought to be an extreme form of a spectrum of clinical conditions, collectively known as ‘ocular surface squamous neoplasias’, which range in severity from mild dysplasia, to carcinoma *in situ* and, ultimately, to invasive carcinoma. Symptoms can range from none, to severe pain and visual loss. Lesions generally arise on exposed areas of the eye, particularly on the nasal side, and treatment involves local excision, or in more severe cases, orbital clearance. Metastases are rare and the prognosis is usually favourable.

Although relatively rare everywhere, conjunctival carcinoma is more frequent in parts of sub-Saharan Africa. Uganda offers a good setting in which to investigate the epidemiology of squamous cell carcinoma of the conjunctiva, because the tumour was relatively frequent there, even before the onset of the HIV epidemic (Templeton, 1973; Wabinga *et al*, 2000). In this report, we examine the association of conjunctival tumours with over 50 possible risk factors, including evidence of infection with HIV-1, HPV-16, -18 and -45 and Kaposi's sarcoma-associated herpesvirus (KSHV; human herpesvirus type 8 [HHV-8]), using data from a case-control study of cancer in adults (Ziegler *et al*, 1997; Newton *et al*, 2001).

## MATERIALS AND METHODS

### Study subjects

The subjects included in these analyses were selected from a large cross-sectional study of risk factors for cancer in Uganda, which included, in total, 2091 individuals with malignancies other than Kaposi's sarcoma. Between August 1994 and February 1998, any adults (aged 15 years and over) with a provisional new diagnosis of incident cancer were eligible for recruitment into the study from all the wards and out-patient clinics of the four main hospitals in Kampala, Uganda: Mulago (including the Uganda Cancer Institute), Nsambya, Mengo and Rubaga. Further details of the methods can be found elsewhere (Ziegler *et al*, 1997; Newton *et al*, 2001). The subjects included in this report are restricted to 60 cases of conjunctival cancer and 1214 controls. Thirty-two people with eye cancers that were not specifically diagnosed clinically as being squamous cell carcinomas, or had a pathological diagnosis of malignancy of uncertain morphology were excluded. The controls comprised people with other incident cancers, excluding those with cancer sites or types that are known to be associated with infection with HIV, HPV or KSHV, or exposure to solar ultraviolet radiation – that is, Kaposi's sarcoma, non-Hodgkin's lymphoma, Hodgkin's disease, cancers of the uterine cervix, anus and penis, and skin cancers (Iarc, 1992, 1995; Beral and Newton, 1998). The control group included men and women with cancers of the oral cavity (57), oesophagus (150), stomach (74), liver (103), breast (178), ovary (67), prostate (56), and other cancer sites or types (405). In addition, 124 patients with a provisional diagnosis of cancer, but who subsequently turned out to have benign tumours, were also included in the control group.

### Recruitment and questionnaire

Interviewers (who were also trained HIV counsellors) approached ward or clinic staff to gain permission to interview potential recruits. If permission was granted, the patient was approached by one of four interviewers and invited to participate in the study. A HIV test was requested and appropriate counselling was given. Patients who had been HIV tested within a month of recruitment and had a medical certificate indicating the test result, were not re-tested. The patient was interviewed about social and demographic factors, and sexual and reproductive history. Where possible, the patient was interviewed by a counsellor of the same sex, and in their native language. Blood was drawn, primarily for the HIV test, but also for storage of serum and leukocytes. Interviewers reported the HIV test results back to the patient, together with post-test counselling. The study was approved by the Committee on Human Research (VA Medical Centre and University of California, San Francisco, CA, USA) and by the Uganda National Council for Science and Technology.

### Laboratory diagnoses

#### Histology

Diagnoses of cancer were established by histology or other laboratory investigation, where possible. Diagnoses made on clinical grounds alone, were reviewed by the investigators (C A-A, JZ, EM and SM). Among the cases, 36 (60%) underwent histological review (all were invasive tumours) and the remainder were diagnosed clinically. Among controls, 63% of tumour diagnoses were verified by laboratory investigation (histology, cytology, blood chemistry or ultrasound examination).

#### HIV-1

HIV-1 serostatus was determined using a single ELISA, the Cambridge Bioscience Recombigen enzyme linked immunosorbent assay (Cambridge, MA, USA). Periodic laboratory quality control assays using blinded standards from the United States Centers for Disease Control (Atlanta, GA, USA) revealed test sensitivity and specificity of 99%. In the initial months of the study, 40–50% of adults declined to have venepuncture, mostly because they were too ill. Therefore, a GACELISA saliva test for HIV was introduced as an option (with sensitivity and specificity similar to the blood test), and the refusal rate dropped to 10%. In total, HIV test results were available on 57 cases and 826 controls. However, for one case and seven controls the result was indeterminate. Any remaining aliquots of sera were stored at −80°C and were later shipped on dry ice to the Laboratoire de Virologie Moléculaire in Tours, France, for HPV testing and to University College London, UK, for KSHV testing.

#### HPV-16, -18 and -45

All assays were performed by a single investigator (LB), who was unaware of each patient's personal characteristics, and diagnosis. HPV VLPs were produced in Sf21 insect cells using recombinant baculoviruses encoding the L1 gene of HPV-16, -18, and -45, according to previously described procedures (Touzé *et al*; 1998; Combita *et al*, 2002a,b). These subtypes were chosen because they are known to be prevalent in tumour specimens from women with cancer of the uterine cervix in Uganda (Bosch *et al*, 1995). Briefly, the HPV L1 genes were first amplified from an HPV DNA-positive biopsy using primers containing *Bgl*II sites. The amplified product was then inserted into pFastBacI to generate HPV L1 VLPs. VLPs were produced according to a procedure used for HPV-16 VLPs (Touzé *et al*, 1998; El Mehdaoui *et al*, 2000). Sf21 cells, maintained in Grace's insect medium supplemented with 10% foetal calf serum (FCS) were infected with the different recombinant baculoviruses at a m.o.i. of 10 and were incubated for 72 h at 27°C. Cells were harvested by centrifugation, re-suspended in PBS containing 0.5% NP40 and allowed to stand at room temperature for 30 min. Cell lysates were then centrifuged at 14 000×g for 15 min at 4°C. The nuclear fractions were further re-suspended in ice cold PBS and sonicated by three 15 s bursts at 60% maximal power (Vibra Cell, Bioblock Scientific, Strasbourg, France). Fractions were then loaded on the top of a preformed CsCl gradient and centrifuged at equilibrium in a Beckman SW28 rotor (20 h, 27 000 r.p.m., 4°C). Gradient fractions were analysed for density by refractometry and were tested for the presence of L1 protein by ELISA. Immunoreactive fractions were finally pooled and pelleted by ultracentrifugation in a Beckman SW 28 rotor (3 h, 28 000 r.p.m., 4°C). VLPs were resuspended in PBS (pH 7.4) and protein content was evaluated using the microBCA kit (Pierce, Touzart et Matignon, France). Each preparation was tested for the presence of VLPs by electron microscopy. For this purpose, VLP preparations were applied to 400-mesh carbon-coated grids, negatively stained with 1.5% uranyl acetate and then examined at a nominal magnification of 50 000 with a Jeol 1010 electron microscope. VLPs were then diluted in PBS and used in the following ELISA tests (Touzé *et al*, 1998; Combita *et al*, 2002a).

Flat-bottomed wells of 96-well microplates (Maxisorp, Nunc, Life Technologies, Eragny, France) were coated overnight at 4°C with 200–400 ng of VLPs (test well) or 200 ng of BSA (control well) in PBS, pH 7.4. Each serum sample was tested twice against each of the seven VLP types and BSA at the same time on the same plate. After washing with PBS, 0.1% Tween 20 and 200 μl of PBS containing 1% newborn bovine serum (NBS, Sigma, St Quentin Favallier, France) were added (2 h at 37°C). The blocking solution was replaced by 100 μl of sera diluted 1 : 20 in 5xPBS-10% NBS and 2% Tween 20, and plates were incubated at 45°C for 60 min. After four washes, bound antibodies were detected with a goat anti-human IgG immunoglobulin (diluted 1 : 5000) conjugated to horseradish peroxidase (Sigma). Following incubation at 45°C for 1 h and four washes, 100 μl of a substrate solution containing ortho-phenylenediamine and H_2_O_2_ was added. After 30 min incubation, the reaction was stopped by addition of 100 μl of 4N H_2_SO_4_, and optical densities (OD) were read at 492 nm with an automated plate reader (BioRad, model 550). For each serum sample the background reactivity found in the BSA coated wells was subtracted from the OD found in each of the HPV–VLP coated wells. Negative values were adjusted to zero. The cut-off values for positivity to HPV-16, -18 and -45 were set up at 0.2 (OD test well minus OD control well) and those with a value of 0.4 or greater were considered to have a high titre of anti-HPV antibodies. In total, 457 samples were available for testing for antibodies against HPV-16 (39 cases and 418 controls). In addition, there were sufficient sera for 453 of those to be tested for antibodies against HPV-18 and -45 (39 cases and 414 controls).

#### KSHV

All assays were performed by a single investigator (DB), who was unaware of each patient's personal characteristics, and diagnosis. A body-cavity-related B-cell lymphoma cell, BCP-1, which is positive for KSHV and negative for Epstein–Barr virus, was used for an indirect immunofluorescence assay to detect IgG antibodies against KSHV antigen (Gao *et al*, 1996; Boshoff *et al*, 1998). Latently infected BCP-1 cells were fixed in 4% paraformaldehyde and were made permeable with 0.2% Triton X-100. Cells were re-suspended in phosphate-buffered saline and fixed on glass slides. The samples were diluted 1 : 100 in phosphate-buffered saline with 3% foetal calf serum. The diluted serum was added to fixed BCP-1 cells and incubated at 22°C for 45 min. After the slides were washed in phosphate-buffered saline with 3% foetal calf serum, rabbit antihuman IgG labelled with fluorescein isothiocyanate (Dako, High Wycombe, UK), diluted 1 : 40 in phosphate-buffered saline with 3% foetal calf serum, was added and the slides were incubated at 22°C for 20 min. The slides were then washed in phosphate-buffered saline without foetal calf serum and screened by ultraviolet microscopy for the nuclear stippling pattern characteristic of antibodies against the latent nuclear antigen of KSHV encoded by orf73 (Gao *et al*, 1996). In total, 416 samples were available for testing for antibodies against KSHV (32 cases and 384 controls).

### Statistical methods

Data were computerised by trained clerks using EPI-INFO-5 software, and statistical analyses were conducted using STATA 7.0 (Dean *et al*, 1990; Stata Corp, 2001). The odds ratio (OR) for each variable in cases versus controls, was estimated with unconditional logistic regression. All odds ratios were adjusted for age (15–24, 25–34, 35–44 and 45+), sex, HIV-1 sero-status and personal income (less than 20 000 Uganda Shillings per year or 20 000+ Shillings). Additional adjustment was made for region of residence (Kampala or elsewhere), because most cases had been identified from out-patient clinics, which tend to draw patients from the local area, whereas controls had been hospital in-patients, who are from a wider area (Ziegler *et al*, 1997). These factors were selected *a priori*, with the exception of personal income, which was included following preliminary analyses. HIV infection is sexually transmitted and is strongly associated with many of the sexual and reproductive variables studied (data not shown). In order to avoid confounding, these factors were examined among HIV seropositive individuals only (there were too few HIV seronegative people to justify analysis). All *P* values are two-sided. Note that numbers of cases and controls in the tables do not always add to the total, because of missing values.

## RESULTS

Among those with conjunctival cancer, 43% (26 out of 60) were men and 57% (34 out of 60) were women. The proportion of all cancers comprising conjunctival carcinoma declined from 9% in those aged 15–24 years to 2% in those over the age of 45 years. Seven per cent of cases and 5% of controls were born in Kampala, the remainder being born outside the capital city (*P*=0.7) and, 41% of cases and 23% of controls reported their current residence as being in Kampala (Table 1

**Table 1:**
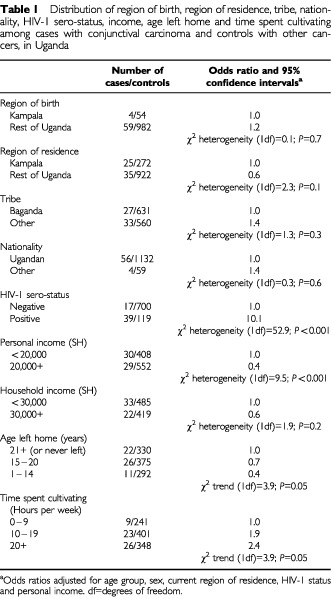
Distribution of region of birth, region of residence, tribe, nationality, HIV-1 sero-status, income, age left home and time spent cultivating among cases with conjunctival carcinoma and controls with other cancers, in Uganda

; *P*=0.13). The seroprevalence of anti-HIV-1 antibodies was 70% among cases and 15% among controls (Odds ratio [OR] 10.1, 95% confidence intervals [CI] 5.2–19.4; *P*<0.001). The risk of conjunctival carcinoma was significantly lower among those with a high personal income (OR 0.4, 95% CI 0.2–0.7; *P*<0.001). For those who left home at ages 21+ years (including those who never left), 15–20 years and 1–14 years, the odds ratio was 1.0 (reference group), 0.7 (0.4–1.5) and 0.4 (0.2–1.0) respectively (*P*_trend_=0.05). Study participants were asked how long each week they spent cultivating, 0–9 h, 10–19 h or 20+ h. The risk of conjunctival carcinoma increased significantly with increasing time spent cultivating (ORs 1.0, 1.9 and 2.4 respectively; *P*_trend_=0.03).

Table 2

**Table 2:**
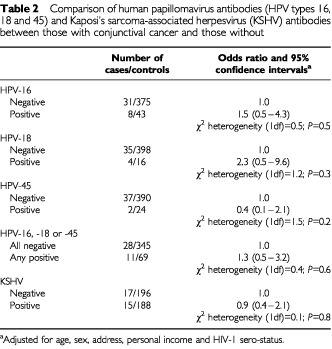
Comparison of human papillomavirus antibodies (HPV types 16, 18 and 45) and Kaposi's sarcoma-associated herpesvirus (KSHV) antibodies between those with conjunctival cancer and those without

shows the results for anti-HPV and KSHV antibodies. The seroprevalence of anti-HPV antibodies in controls was 10% for HPV-16 (43 out of 418), 4% (16 out of 414) for HPV-18 and 6% (24 out of 414) for HPV-45. The corresponding results for those with conjunctival cancer were 21% (eight out of 39), 10% (four out of 39) and 5% (two out of 39) respectively. However, after adjustment for age, sex, address, HIV status and personal income, there were no statistically significant associations between the presence of anti-HPV-16, -18 and -45 antibodies and the risk of conjunctival carcinoma. Results for each HPV subtype were also calculated according to a measure of the antibody titre: the optical densities at each level correspond to less than 0.2 for negative, 0.2−0.39 for medium titre and 0.4 or above for high titre. The numbers of cases and controls with anti-HPV antibodies to subtypes -18 and -45 were too few to yield any significant results. The results for anti-HPV-16 antibodies at each measure of titre were 1.0 (HPV-16 antibody negative, based on 31 cases and 375 controls), 0.7 (0.2–2.9; medium titre, based on four cases and 31 controls) and 6.3 (1.2–33.4; high titre, based on four cases and 12 controls; *P*_trend_=0.2). Only 15 people had anti-HPV antibodies to more than one tested HPV subtype (two cases and 13 controls) and there was no significant excess risk of the tumour in these individuals, as compared to those who were considered to be negative for all three subtypes (OR 0.6, 95% CI 0.1–4.3). In relation to Kaposi's sarcoma-associated herpesvirus, the seroprevalence of anti-KSHV antibodies was 47% (15 out of 32) among cases and 49% (188 out of 384) among controls (OR 0.9, 95% CI 0.4–2.1; *P*=0.8).

Further results are provided in Appendices 1–4. Results for other social and demographic factors, none of which was associated with an increased risk of conjunctival carcinoma, are shown in Appendix 1. Factors, other than income, that might characterise wealth and social status in Uganda, such as ownership of livestock and travel away from home, are shown in Appendix 2. None of these variables were associated with conjunctival carcinoma. Appendix 3 shows results for other possible exposures. Those who reported having had a blood transfusion in the past were less likely to be cases (OR 0.4, 95% CI 0.2–0.9; *P*=0.02). Results for sexual and reproductive variables among HIV seropositive people only, are shown in Appendix 4. A self reported history of having either given or received gifts in exchange for sex was associated with an increased risk of the tumour (OR 3.5, 95% CI 1.2–10.4; *P*=0.03), although this may have been a chance finding as there was no statistically significant association with any other sexual or reproductive variable, including the reported number of lifetime sexual partners (*P*=0.4).

## DISCUSSION

Here, we report an investigation of the epidemiology and aetiology of conjunctival squamous cell carcinoma, examining over 50 risk factors for the tumour. Conjunctival cancer affects relatively young individuals of both sexes and is strongly associated with HIV infection and poverty. In addition, the risk of the disease decreases with decreasing age at which an individual leaves home and with increasing time spent cultivating, although both results are of borderline statistical significance. Furthermore, significance tests were conducted on over 50 risk factors, greatly increasing the likelihood of significant results arising by chance alone. The role of HPV-16, -18 and -45 requires further evaluation, but KSHV does not appear to be associated with conjunctival carcinoma.

The study reported here is subject to the potential problems of incomplete diagnostic verification and HIV testing. Laboratory verification of cancer diagnosis (for example, by histology, cytology or blood chemistry) was available on 60% of cases (all of which were invasive tumours) and 63% of controls. Typical of studies in developing countries where laboratory services are limited, this proportion compares favourably with other cancer series reported from Africa (Bassett *et al*, 1995; Newton *et al*, 1996a; Wabinga *et al*, 2000). Another potentially important source of bias is the high proportion of adults who were not tested for HIV in the early stages of the study. However, the age and sex-specific seroprevalence rates of HIV infection among those tested is broadly similar to that found in studies of HIV seroprevalence in Uganda (STD/AIDS Control Programme, Uganda, 1997). Also, the introduction of a saliva test resulted in a dramatic reduction in refusals, but little change in HIV seroprevalence rates, suggesting that patients who were refusing to have a HIV test were unwilling to have blood taken, rather than unwilling to learn their HIV serostatus. The association of conjunctival cancer with HIV infection has been reported in a general paper on HIV and cancer among residents of Kampala, and the odds ratio was the same as that from the current study of all patients who were seen at hospitals in Kampala (Newton *et al*, 2001). A proportion of individuals in the study were also tested for antibodies to three HPV subtypes and to KSHV infection. This additional testing occurred in all those for whom enough stored sera were available and (as people are unaware of their own status with respect to infection), is therefore an unbiased sample.

Another potential source of bias arises from the fact that most (but not all) cases had been identified from out-patient clinics, which tend to draw patients from the local area, whereas controls had been hospital in-patients, who are from a wider area. Despite adjusting for region of residence in the analyses, the possible impact of this on the results, is difficult to assess.

Case reports of squamous cell carcinoma of the conjunctiva in human immunodeficiency virus (HIV) infected men in the USA and France, coupled with a marked increase in the numbers of tumours being seen by ophthalmologists in at least two African centres (which mirrored the increases seen for Kaposi's sarcoma), led to the suggestion of an association with HIV (Winward and Curtin, 1989; Ateenyi-Agaba, 1995; Kestelyn *et al*, 1990; Kim *et al*, 1990; Denis *et al*, 1994). Among those with HIV, the lesions often affect young adults, in a fashion reminiscent of Kaposi's sarcoma in HIV-seropositive people (Ateenyi-Agaba, 1995; Kestelyn *et al*, 1990; Waddell *et al*, 1996). This study, together with others from Africa and the USA indicate about a 10-fold increased risk of the tumour in HIV infected, compared to HIV uninfected individuals (Ateenyi-Agaba, 1995; Kestelyn *et al*, 1990; Goedert and Coté, 1995; Newton *et al*, 1995, 2001; Waddell *et al*, 1996). Indeed, the spread of HIV in Uganda probably accounts for much of the approximately eight-fold increase in incidence of conjunctival carcinoma observed there since the 1960s (Wabinga *et al*, 2000). Using standard equations for case–control studies, the population attributable fraction is about 60%: almost two-thirds of cases would not occur in the absence of HIV infection (dos Santos Silva, 1998). However, the frequency of conjunctival carcinoma is not such that it is yet a particularly common manifestation of HIV disease (Piot *et al*, 1992).

The mechanism whereby HIV infection increases the risk of conjunctival cancer is not clear. There is no evidence that it is directly oncogenic and the impact on cancer risk is most likely to be mediated via immunosuppression, as is the case for other HIV-associated cancers (Beral and Newton, 1998; Newton *et al*, 1999). It is not known if the risk of conjunctival squamous cell carcinoma is increased in other immunosuppressed groups, such as transplant recipients, because the tumour is very rare in parts of the world where tissue transplantation occurs. However, there is a case report of a conjunctival cancer in a patient with malignant lymphoma on immunosuppressive chemotherapy (Kushner and Mushen, 1975). In general, HIV infection is thought to increase cancer risk by facilitating the action of other oncogenic viruses, such as KSHV, the principal cause of Kaposi's sarcoma. In relation to conjunctival cancer however, we found no evidence in these data of an association with KSHV.

Several types of squamous carcinoma are associated with human papillomavirus (HPV) infection, most notably cancer of the uterine cervix, induced primarily by HPV-16, -18 and others. Squamous carcinoma of the skin has also been associated with HPV-5 and -8 in immunosuppressed individuals (Iarc, 1995). Evidence for an association between human papillomavirus and squamous cell carcinoma of the conjunctiva is conflicting, although bovine papillomaviruses are thought to cause conjunctival carcinomas in cattle (Iarc, 1995). The presence of human papillomavirus DNA (HPV; predominantly types 16, but also other types) in human ocular surface squamous neoplasias, including invasive carcinomas, has been reported in some studies, but not others (reviewed by Newton, 1996). This is the first study to look for an association between anti-HPV antibodies (types -16, -8 and -45) and the risk of conjunctival carcinoma. The seroprevalence of antibodies against HPV-18 and -45 was too low to make reliable conclusions. Results for anti-HPV-16 antibodies were suggestive of an association among individuals with high titres, but the data presented here are too few to draw valid conclusions and studies of larger numbers of cases are required.

The HPV assays used in this study are based on the expression of L1 major capsid proteins of HPV-16, -18 and -45 in insect cells by using recombinant baculoviruses (Touzé *et al*, 1998). The resulting virus-like particles (VLPs), which appear similar to empty virions, can be used in serological studies to test for type specific immunological responses to viral capsid proteins, although there is evidence that a particular assay may cross react with related HPV subtypes (Combita *et al*, 2002b). Presence of anti-VLP antibodies is an indicator of past and current infection (Kirnbauer *et al*, 1994; Le Cann *et al*, 1995; Wideroff *et al*, 1995; Dillner *et al*, 1996). The utility of such assays has been demonstrated in previous studies of anti-HPV-16 antibodies in relation to the risk of cancer of the uterine cervix (Lehtinen *et al*, 1996; Dillner *et al*, 1997; Shah *et al*, 1997; Vonka *et al*, 1999; Hisada *et al*, 2001). Results from this study on the relationship between anti-HPV-16 antibodies and the risk of cancer of the uterine cervix are broadly comparable to those reported before and will be the subject of a separate report.

Although relatively rare everywhere, conjunctival carcinoma is more frequent in sub-Saharan Africa and other tropical areas than in temperate countries, leading to the hypothesis that it may be associated with exposure to solar ultraviolet radiation (Templeton, 1973; Clear, 1979). Geographical studies support this premise, in that the incidence of squamous carcinoma of the eye increases as exposure to ambient solar ultraviolet radiation increases. Levels of solar radiation are higher towards the equator and the incidence of conjunctival cancer increases by about 50% for each 10° decline in latitude (Newton *et al*, 1996b; Sun *et al*, 1997). In addition, a single case–control study reported that the risk of ocular surface epithelial dysplasias is greatest in those reporting a past history of skin cancer, which is known to be caused by exposure to solar ultraviolet radiation (Lee *et al*, 1994). Time spent cultivating may indicate the time an individual spends in direct sunlight, and so the increasing risk with increasing time cultivating may reflect exposure to ultraviolet radiation. This variable may also reflect exposure to dust or dirt, and it has been hypothesised that ocular trauma could facilitate development of the tumour (Templeton, 1973; Margo and Groden, 1986). The lower risk among people who leave home at an earlier age may reflect migration to towns for work, where exposure to solar ultraviolet radiation could be less. The extent to which the higher risk among those of low income is measuring the previously mentioned exposures is not clear. The fact that cases are less likely than controls to have had a blood transfusion is probably an artefact of the control selection. Cases were generally seen as out-patients, whereas controls with other cancers were generally hospital in-patients. The latter may therefore be more likely to have had a transfusion as part of their medical care.

In summary, the current study gives independent support for the strong epidemiological evidence that solar ultraviolet radiation is an important cause of squamous cell carcinoma of the conjunctiva. Another established risk factor is HIV infection, although the mechanism whereby it increases the risk of conjunctival cancer is not clear. We find little evidence supporting the possible role of sexually transmitted forms of HPV in the aetiology of conjunctival cancer, but larger studies are required.

## Figures and Tables

**Table 3:**
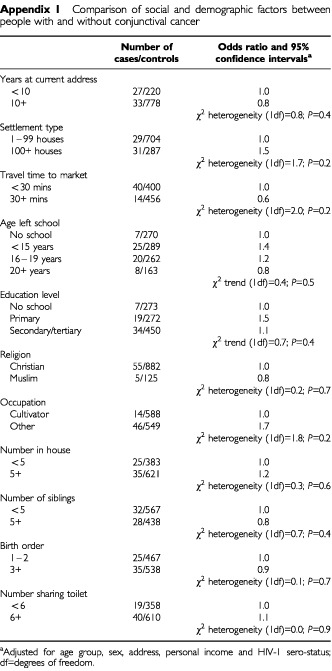
Comparison of social and demographic factors between people with and without conjunctival cancer

**Table 4:**
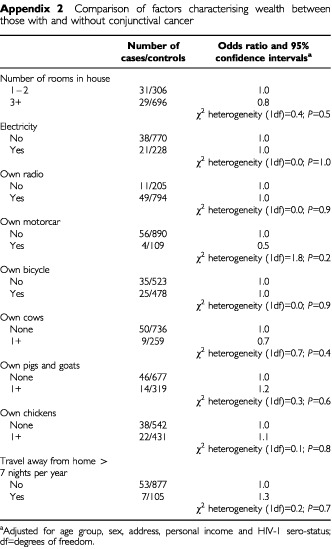
Comparison of factors characterising wealth between those with and without conjunctival cancer

**Table 5:**
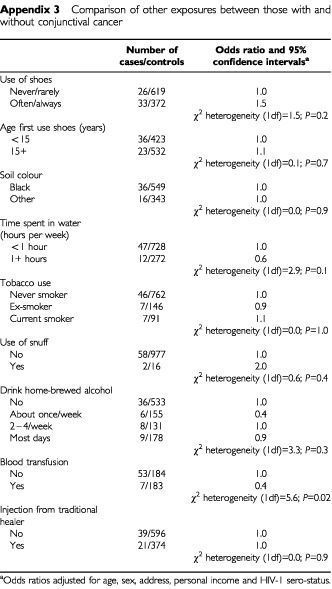
Comparison of other exposures between those with and without conjunctival cancer

**Table 6:**
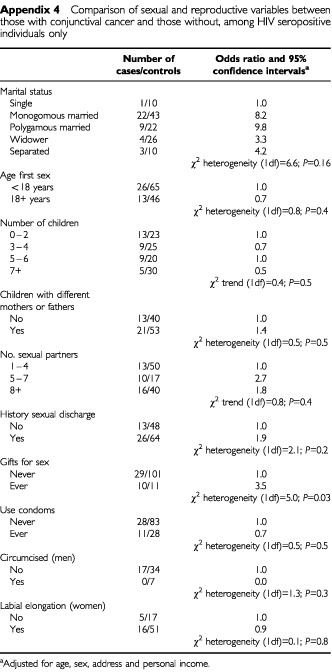
Comparison of sexual and reproductive variables between those with conjunctival cancer and those without, among HIV seropositive individuals only

## Appendix 1

Table 3

## Appendix 2

Table 4

## Appendix 3

Table 5

## Appendix 4

Table 6
