# The epidemiology of conjunctival squamous cell carcinoma in Uganda

**DOI:** 10.1038/sj.bjc.6601128

**Published:** 2003-07-15

**Authors:** R Newton, J Ziegler, C Ateenyi-Agaba, L Bousarghin, D Casabonne, V Beral, E Mbidde, L Carpenter, G Reeves, D M Parkin, H Wabinga, S Mbulaiteye, H Jaffe, D Bourboulia, C Boshoff, A Touzé, P Coursaget, the Uganda Kaposi's Sarcoma Study Group

## Abstract

**Correction to**: *British Journal of Cancer* (2002) **87**, 301–308. doi:10.1038/sj.bjc.6600451

In the sixth line of the abstract, the confidence interval in relation to personal income is incorrect. It should read:

OR=0.4, 95% CI 0.3–0.7; *P*<0.001.

It is correctly stated later in the text.

The authors apologise for any inconvenience this may have caused.

